# Evaluating the Combined Effect of a Choline Kinase Inhibitor and Temozolomide Therapy in a Mouse Model of Glioblastoma Using ^1^H MR Spectroscopy and IVIM‐DWI

**DOI:** 10.1002/nbm.70113

**Published:** 2025-08-04

**Authors:** Tareq Alrashidi, Sourav Bhaduri, Elisabeth Non Gash, Mohesh Moothanchery, Christopher Ball, Mahon L. Maguire, Lorenzo Ressel, Harish Poptani

**Affiliations:** ^1^ Centre for PreClinical Imaging University of Liverpool Liverpool UK; ^2^ Institute for Advancing Intelligence (IAI), TCG CREST Kolkata India; ^3^ Academy of Scientific and Innovative Research (AcSIR) Ghaziabad India; ^4^ Department of Veterinary Anatomy Physiology and Pathology University of Liverpool Chester UK

**Keywords:** cancer, GBM, glioblastoma, IVIM, microstructure, MR spectroscopy, treatment response

## Abstract

This study evaluated the therapeutic efficacy of combining a choline kinase alpha (ChoK*α*) inhibitor, MN58b, and temozolomide (TMZ) in a syngeneic GL261 glioblastoma (GBM) mouse model. It used MR spectroscopy (MRS) and intravoxel incoherent motion diffusion‐weighted imaging (IVIM‐DWI) to assess metabolic and microstructural changes within the tumor. Fifty‐two C57BL/6 mice had GL261 cells implanted intracranially and were divided into four groups: saline control, MN58b, TMZ, and MN58b + TMZ (*n* = 16, 14, 11, and 11, respectively). Treatments were administered for 5 days, starting 10 days post‐implantation. MRI scans (T_2_‐weighted, MRS, IVIM‐DWI) were performed at baseline, during treatment (Day 3), and post‐treatment (Day 6). The histological analysis evaluated the tumor mitotic index and caspase‐3 expression. Combination therapy with MN58b + TMZ significantly reduced tCho/NAA, Lip + Lac/tCr, and mI/tCr, suggesting decreased phosphocholine synthesis and tumor proliferation. IVIM‐DWI showed a significant increase in diffusion coefficient (D) values, indicating reduced cell density. Metabolic changes detected by ^1^H MRS were observable as early as Day 3 post‐treatment initiation, preceding microstructural alterations detected by IVIM‐DWI at Day 6. This suggests that MRS biomarkers may serve as early indicators of treatment response, facilitating timely therapeutic decisions. Histology confirmed a significantly lower mitotic index relative to control tumors in the combination treatment group. Significantly prolonged survival with combination therapy was noted relative to other groups. However, the tumor volumes were not significantly different between groups. Combination therapy targeting ChoK*α* and cellular proliferation with MN58b and TMZ outperformed individual treatments for GBM, warranting further exploration. The integration of MN58b with the current standard of care (TMZ + RT) might further enhance the therapeutic outcomes. MRS and IVIM‐DWI demonstrate potential utility as non‐invasive imaging markers for treatment monitoring.

## Graphical abstract

Significant decreases in tCho/NAA along with increased apparent diffusion coefficient were observed in response to a combinatorial effect of temozolomide and choline kinase inhibitor in a mouse model of glioblastoma. These changes corresponded to reduced mitoses and occurred prior to changes in tumour volume, indicating imaging and spectroscopy could be used as early markers of treatment response.

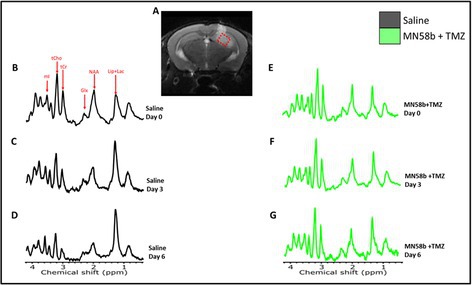

**Abbreviations**
ADC: apparent diffusion coefficient
ANOVA: analysis of variance
BBB: blood–brain barrier
ChoKα: choline kinase alpha
CKIs: choline kinase inhibitors
CRLB: Cramér–Rao lower bound
CT: computed tomography
DAB: diaminobenzidine
DW‐MRI: diffusion‐weighted MRI
EGFR: epidermal growth factor receptor
FOV: field of view
GBM: glioblastoma
GLUT3: glucose transporter 3
Glx: glutamate–glutamine
GSCs: glioma stem cells
HLSVD: Hankel Lanczos singular value decomposition
IHC: immunohistochemistry
IVIM: intravoxel incoherent motion
Lip + Lac: lipid pluslactate
MGMT: methyl guanine‐DNA methyl transferase
mI: myo‐inositol
MRI: magnetic resonance imaging
MRS: magnetic resonance spectroscopy
NAA: N‐acetylaspartate
NF1: Neurofibromin 1
NIVO: nivolumab
OS: overall survival
PC: phosphocholine
PDGFRA: platelet‐derived growth factor receptor A
PRESS: point‐resolved spectroscopy
PUFA: polyunsaturated fatty acid
QUEST: quantum estimation
RANO: response assessment in neuro‐oncology
RARE: rapid acquisition with relaxation enhancement
ROI: region of interest
RT: radiation therapy
TCA: tricarboxylic acid
tCho: total choline
tCr: total creatine
TE: time of echo
TME: tumor microenvironment
TMZ: temozolomide
TR: time of repetition
VEGF: vascular endothelial growth factor

## 1 Introduction

Glioblastomas (GBMs) are highly aggressive brain tumors associated with poor prognoses [1]. Patients with GBM have a median survival time of less than 15 months following diagnosis [2]. Typically, a mass‐occupying lesion in the brain demonstrating contrast enhancement on computed tomography (CT) or magnetic resonance imaging (MRI) is observed in a majority of patients with GBM [3, 4]. The current standard of care for GBM entails maximal safe resection of the tumor followed by concurrent and adjuvant temozolomide (TMZ) along with radiation therapy (RT). This regimen has significantly improved 2‐year overall survival (OS) from 10.9% with RT alone to 27.2% with TMZ + RT [5, 6].

TMZ, a second‐generation alkylating agent, efficiently crosses the blood–brain barrier (BBB) and exhibits excellent oral bioavailability. It's anticancer activity is mediated by DNA methylation. Methylation of the O6‐methylguanine‐DNA methyltransferase (MGMT) promoter reduces MGMT expression, increasing chemosensitivity and decreasing DNA repair capability. Despite this, MGMT‐methylated tumors eventually develop resistance to TMZ with inevitable tumor recurrence, contributing to poor prognosis [7, 8]. As such, novel drugs are desperately needed to overcome treatment resistance. However, drugs targeting angiogenesis, such as bevacizumab, have not improved OS outcomes in patients with GBMs [9].

A hallmark of cancer is altered cellular metabolism, with increased aerobic glycolysis being one of the most frequently identified aberrant metabolic changes [10, 11]. In heterogeneous cancers like GBM, metabolic plasticity has also been observed [12, 13]. A previous study reported that ^13^C‐glucose infusion during operative resection of a cerebral tumor resulted in pyruvate dehydrogenase‐driven oxidation of ^13^C‐pyruvate into the tricarboxylic acid (TCA) cycle [12]. This highlights the fluctuating metabolic nature within the tumor microenvironment (TME) depending on glucose availability. Because reprogramming metabolic activity contributes to oncogenesis, targeting metabolic processes may represent a promising strategy for new cancer therapeutics [14].

Recent research indicates that glioma stem cell's (GSCs) high glucose demand promotes their survival by upregulating glucose transporter 3 (GLUT3) [15]. Increased glycolysis and overexpression of GLUT3 and hexokinase II are linked to poor prognosis in GBM [15, 16]. Furthermore, molecular subtypes of GBM exhibit changes in genes that include epidermal growth factor receptor (EGFR), Neurofibromin 1 (NF1), and platelet‐derived growth factor receptor A (PDGFRA), which affect various metabolic pathways.

In gliomas, EGFR overexpression drives the upregulation of choline transporters and enhances choline kinase α (ChoKα) activity, leading to increased choline uptake and metabolism. This EGFR‐mediated metabolic reprogramming results in elevated phosphocholine (PC) and total choline (tCho) [17, 18]. Hence, targeting metabolic pathways could also offer a way to overcome therapy resistance [5, 19].

Abnormal choline metabolism has been recognized as a sign of malignancy [20]. Upregulation of choline kinase alpha (ChoK*α*) has transformed normal tissue into a neoplastic phenotype [17]. The discovery of upregulated ChoK activity has driven the development of new anticancer therapies to inhibit this enzyme. Several choline kinase inhibitors (CKIs) have shown antiproliferative effects [21, 22]. MN58b, a first‐generation CKI, has demonstrated efficacy in vitro and in vivo [23]. However, a recent study by Bhaduri et al. did not show any significant therapeutic benefit despite metabolic alterations in tCho levels in the GL261 model treated with the CKI, JAS239 [24]. These findings suggest that while CKIs may effectively modulate tumor metabolism, their ability to reduce tumor growth or improve survival outcomes may be limited in aggressive GBM.

TMZ is the standard chemotherapy for GBM; however, resistance mechanisms often limit its efficacy, including the DNA repair enzyme O6‐methylguanine‐DNA methyltransferase (MGMT) and other DNA repair pathways. MN58b is a ChoKα inhibitor that disrupts choline metabolism, an upregulated pathway in GBM. Our rationale is that combining TMZ with MN58b will target both DNA integrity and membrane phospholipid metabolism, potentially overcoming resistance and addressing the need for more effective treatments for GBM. This might be a more effective approach as targeting angiogenesis, such as with bevacizumab, has not improved OS outcomes in patients with GBMs.

MRI is widely used to monitor tumor response to therapy in clinical and preclinical settings. However, traditional MRI methods cannot always distinguish treatment response from progression, as both may present similar imaging features [25, 26]. Changes in tumor microstructure and metabolism may precede volumetric changes and are not always detected with conventional MRI [27, 28]. We assessed the efficacy of this combination therapy in a syngeneic GBM mouse model employing magnetic resonance spectroscopy (MRS) for evaluating metabolic alterations and diffusion‐weighted MRI (DW‐MRI), specifically intravoxel incoherent motion (IVIM) imaging to monitor microstructural changes within the tumor.

## 2 Materials and Methods

University of Liverpool's Animal Welfare and Ethical Review Board authorized all animal experiments that followed ARRIVE principles, and the study was approved by the Home Office in compliance with the UK Animals (Scientific Procedures) Act 1986, as revised in 2012.

Two hundred fifty thousand GL261 cells (ATCC, USA) were transplanted intracranially into male C57BL/6 mice (age: 8–12 weeks, weight: 20–23 g, *N* = 52). A rectal probe and a respiratory pillow were used to measure the animal's body temperature and breathing rate after having been anesthetized with 2% isoflurane in O_2_. The animal was secured employing a stereotaxic frame, and a burr hole was drilled 2.0 mm posteriorly from bregma and 1.5 mm to the right. GBM cells suspended in 5 *μ*L of serum‐free media were injected into the right brain cortex at a depth of 2 mm from the surface.

### 2.1 Drug Treatment of Mice Bearing GL261 GBM

All mice successfully developed tumors and were randomly divided into four groups: saline control (*n* = 16), MN58b (*n* = 14), TMZ (*n* = 11), and a combination therapy group (MN58b + TMZ, *n* = 11). All animals received a single dose every day for five consecutive days. Treatment commenced when tumors exceeded 2 mm in diameter, typically 10 days post‐implantation. The control group received intraperitoneal injections of saline, the MN58b group received 4 mg/kg of MN58b intraperitoneally [29], the TMZ group received 50 mg/kg TMZ orally via gavage [30], and the combination (MN58b + TMZ) therapy group received both treatments as above.

### 2.2 MR Scanning

A 9.4T Bruker 94/20USR scanner with 440 mT/m imaging gradients was used for all imaging studies (Bruker BioSpin, Germany). An 86 mm birdcage coil was used to transmit the signal, and a 4‐channel phased array surface coil (Bruker BioSpin, Germany) was used to receive the signal. Animals were anaesthetized as described above, and body temperature was maintained using a heated water blanket; respiration and body temperature were monitored throughout scanning. To monitor tumor growth, T_2_‐weighted (RARE) images were acquired (TR/TE/echo spacing [ESP] = 3497/33/11 ms, 2 averages, 33 slices, 0.3 mm slice thickness, FOV = 25 × 25 mm^2^, 256 × 256 matrix, RARE factor = 8, BW = 42 kHz). Animals began receiving treatment once tumors were > 2 mm in diameter, typically 10 days post‐implantation. Animals were scanned at baseline (Day 0), during therapy (Day 3), and at the end of treatment (Day 6). T_2_‐weighted images, single voxel water suppressed and unsuppressed PRESS spectra, and IVIM‐DWI images were acquired at each time point. A 2 × 2 × 2 mm^3^ PRESS voxel was placed within the tumor, field map‐based shimming was performed (Mapshim, Bruker; linewidth < 20 Hz), and a ^1^H spectrum was acquired (TR/TE = 2000/16.5 ms, 200 averages, 2048 points, spectral width = 4401 Hz, VAPOR water suppression, acquisition time = 6 min 40 s). IVIM DW‐MRI was acquired covering the tumor volume employing a segmented echo‐planar imaging (EPI) readout based spin echo sequence with 5 B_0_ images and 13 b‐values (20, 30, 40, 50, 60, 80, 140, 230, 350, 570, 840, 1120, 1460 s/mm^2^) in 3 orthogonal directions (TR/TE = 1800/24.67 ms, 6 segments, FOV = 20 × 20 mm^2^, 96 × 96 matrix, 1.0 mm slice thickness, 1 mm slice gap, 8 slices, 2 averages, diffusion gradient duration/separation = 4.5/10.6 ms, acquisition time = 15 min 50 s). All images were acquired in the axial plane.

## 3 Data Analysis

Tumor volumes were measured from the T_2_‐weighted images utilizing ITK‐Snap (Version 4.2.0) (www.itksnap.org). Contouring of the tumor was performed manually by drawing the tumor boundaries on each slice. The areas of the contoured regions on each slice were added, and the result was multiplied by the thickness of each slice for calculating the overall tumor volume.

All spectral processing was carried out using jMRUI 5.2 (www.jmrui.eu) [31]. The residual water peak at 4.7 ppm was removed employing a Hankel Lanczos singular value decomposition (HLSVD) filter with model order 25 prior to measuring water‐suppressed spectroscopic data [32, 33]. A 5 Hz Lorentzian line‐broadening function and zero filling to 4196 points were employed for apodization. The QUEST method was applied in jMRUI for fitting metabolite peaks [34, 35]. A basis set of the metabolites including “total choline (tCho) 3.2 ppm, N‐acetyl‐aspartate (NAA) at 2.01 ppm, glutamate‐glutamine (Glx) 2.35 and 3.74ppm, lipid/lactate (Lip+Lac) 1.3 ppm, total creatine (tCr) 3.03ppm, myo‐inositol+Glycine (mI+Gly) 3.56ppm and other metabolites including glucose, phosphocreatine (at 3.9ppm), lipid (at 0.9ppm), alanine, glutathione, aspartate, glycerophosphocholine, GABA, acetate, phosphorylcholine, N‐acetylaspartylglutamate, scyllo‐inositol and taurine” were simulated employing NMR scope tool within jMRUI [30]. The peaks at 1.3 and 0.9 ppm correspond to macromolecules, including lipids. Water amplitude was determined by fitting the water resonance obtained from unsuppressed water spectra. The following equation was used for scaling spectra to unsuppressed water signals, considering the correction factors for T_1_ and T_2_ of water [36].

(1) $$\text{Correction factor}=1-e^{\frac{-\mathrm{TR}}{T_{1,\text{water}}}}e^{\frac{-\mathrm{TE}}{T_{2,\text{water}}}}$$

Using the T_1*,water*_ = 2097 *±* 68 ms and T_2*,water*_ = 42 *±* 1.6 ms as reported for rat brain at 9.4T [37]. Cramér–Rao lower bound (CRLB) criteria were employed to evaluate the quality of the fitting. CRLB/amplitude < 20% was used in discriminating well‐fitted from poorly fitted metabolites. All metabolites within the spectrum that fulfilled this criterion were then employed for data analysis, excluding the ones that did not. A representative example of the MRS fitting performance is shown in Figure S1, adapted from our previously published work [24]. Several factors confound absolute metabolite quantification in tumors, like significant heterogeneity in T1 and T2 relaxation times within gliomas due to necrosis, edema, and disrupted vasculature; variability in water content and cellular density; partial volume effects due to infiltrative tumor margins. These confounders introduce substantial uncertainty in water‐scaling–based absolute quantification. Metabolite ratios were therefore measured with respect to tCr, a commonly used internal reference in MRS due to its relatively stable levels in normal and tumor tissues, allowing for consistent comparisons.

## 4 Parameter Calculation

All images were processed with an in‐house pipeline employing MATLAB 2021a (Mathworks, USA). The data were analyzed and fitted on a pixel‐by‐pixel basis using the Levenberg–Marquardt algorithm [38].

IVIM describes the signal intensity from an image acquired with a b‐value where *b* = 0 (S_0_) and *b* > 0 s/mm^2^ (S_*b*_):

(2) $$\frac{S_{b}}{S_{0}}={\mathrm{fe}}^{-bD^{*}}+\left(1-f\right)e^{-\mathrm{bD}}$$

where *b* is the magnitude of the applied diffusion gradient in s/mm^2^, D* is the pseudo diffusion coefficient in mm^2^/s due to effects of blood flow, *D* is the diffusion coefficient in mm^2^/s, and *f* is the fraction of volume occupied by blood flowing in the voxel [39]. When *b* > 200 s/mm^2^, the value of *fe* ^−bD*^ can be neglected, and the equation can be simplified to [40]:

(3) $$\frac{S_{b}}{S_{0}}=\left(1-f\right)e^{-\mathrm{bD}}$$

A linear model can determine *D* from data at b‐values above > 200 s/mm^2^ with zero intercept. As shown below, *S*_*int*_ determines the perfusion fraction *f* [41].

(4) $$f=1-\frac{S_{\mathrm{int}}}{S_{0}}$$

The data were then fitted to Equation (2) using the value of *D* obtained from Equation (3) as a fixed value and *f* calculated from Equation (4) as an initial value. The initial value for *D* ^*^ was set to 0.01 mm^2^/s, as described previously [41]. The values of *D* ^*^ and *f* were then estimated from the nonlinear fit. The full biexponential model in Equation (2) was fit to the entire dataset (*b* = 0–1460 s/mm^2^). All data were fitted using a nonlinear Levenberg–Marquardt method.

Apparent diffusion coefficient (ADC), which represents a combination of diffusion and perfusion effects, was estimated from the mono‐exponential fit of the diffusion signal decay for all b‐values using:

(5) $$\frac{S_{b}}{S_{0}}=e^{-b.\mathrm{ADC}}$$

In‐house pipeline employing MATLAB 2021a was used to estimate the voxel‐wise metric values of IVIM‐DWI (ADC, *D*, D*, and *f*). The IVIM‐DWI and T_2_‐weighted images were used to locate the slices displaying the tumor, and the region of interest (ROI) was contoured manually, covering the whole tumor. All ROIs for IVIM parameter estimation (*D*, D*, and *f*) were manually delineated on the b=0 image from the EPI sequence. Separately, ROIs were delineated on the T2‐weighted image to measure the tumor volume.

## 5 Histology and Immunohistochemistry (IHC)

Predefined endpoints, including > 20% body weight loss, reduced mobility, lack of grooming, or tumor volume exceeding 6 mm in diameter, were used as endpoints to sacrifice the animal. Animals were monitored daily and, upon reaching any endpoint, were sacrificed in accordance with approved institutional animal care protocols. Following Day 6 imaging, three animals from each group were sacrificed via an intraperitoneal injection of an overdose of 3 mL/kg pentobarbital sodium (Euthatal, Merial Animal Health Ltd., Harlow, UK). The aorta beneath the diaphragm was severed by making an incision into the abdomen along the mid‐ventral line. A 25‐gauge needle attached to an extension tube was inserted into the heart's left ventricle and secured with a clamp. The heart was initially perfused with 30 mL PBS, then 30 mL 4% formalin (Sigma‐Aldrich, St. Louis, MO). Brains were collected following fixation and then immersed in 4% formalin for fixation. Brains were embedded in paraffin and sectioned into approximately 2‐mm thick transverse sections at the tumor site. Hematoxylin and eosin staining was performed on 4 *μ*m thick slices containing tumors and adjacent brain tissue. An average number of mitotic cells was computed per high power field (HPF: 400× magnification; ocular FN: 22; objective 40×/0.65 HPF) representing randomly selected HPFs per tumor to calculate the mitotic index, which was evaluated from five regions at the interface of the tumor and the surrounding brain (infiltrating margin).

For IHC, sections were dewaxed and subjected to antigen retrieval in Dako PT buffer high/low pH (Agilent Technologies Ltd., Stockport, UK) using a computer‐controlled antigen retrieval workstation (PT Link; Agilent Technologies Ltd) for 20 min at 98*°*C. Sections were then stained in an automated immunostainer (Link 48; Agilent Technologies Ltd), using primary antibodies against caspase‐3 (rabbit monoclonal anti‐caspase‐3, 5A1E 1:100). This was followed by a 30 min incubation at room temperature with the secondary antibody and polymer peroxidase‐based detection system (Anti Rabbit Envision Flex+, Agilent Technologies Ltd). The reaction was visualized with diaminobenzidine (DAB—Agilent Technologies Ltd). Consecutive sections incubated with rabbit subclass‐matched unrelated monoclonal antibodies served as negative controls. A distinct brown cytoplasmic stain represented the positive reaction.

For IHC quantification, caspase3‐stained slides were scanned using a Leica Aperio CS2 (Leica Microsystem Limited, Solihull, UK) scanner at 20× magnification. Whole‐slide image analysis was undertaken using QuPath software [42] as previously described [43]. Briefly, the tumor area was manually annotated as the ROI excluding the healthy brain. Positive IHC cells were identified by the presence of a brown colour, resulting from the 3,3′diaminobenzidine (DAB) reaction. Positive cell detection parameters were manually optimized based on sample areas from all the cases, and analysis was run for all cases. Two different masks were used to categorize the cells according to staining: negative (blue) and positive (red) (Figure S2). The software then automatically counted total positive cells and % of positive cells within the ROI.

## 6 Statistical Analysis

Analysis of variance (ANOVA) was performed to evaluate differences among treated groups and the control group (% change in tumor volume, IVIM‐related parameters, and metabolite ratios on Days 3 and 6 scans with respect to baseline [Day 0] values). Bonferroni's post hoc test was utilized for adjusting the *p*‐value, accounting for the total number of comparisons made. The overall percentage survival was estimated using Kaplan–Meier curves. MATLAB and Prism Version 10.2.0 (GraphPad, USA) were employed for all analyses. A value was considered statistically significant if *p* < 0.05.

## 7 Results

### 7.1 Treatment Effect on GL261 Mouse Glioma Growth

**FIGURE 1:**
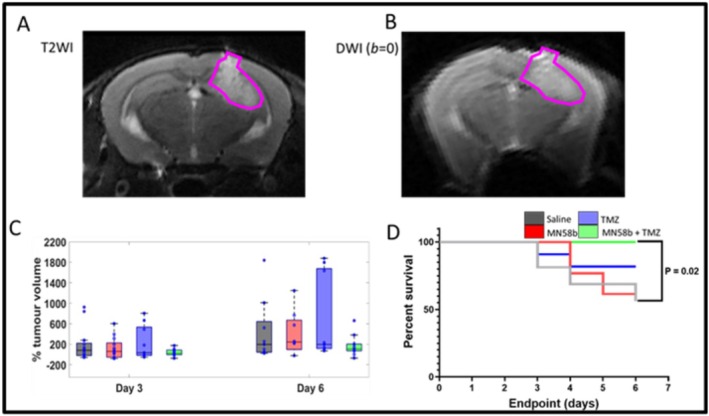
(A–D) Treatment effect on GL261 GBM growth. (A) T_2_‐weighted image showing hyperintense tumor relative to the surrounding tissue. (B) Diffusion‐weighted image (*b* = 0 s/mm^2^) showing the high signal intensity of the tumor compared with the rest of the brain. (C) Percent change in tumor volume relative to Day 0 over treatment time for all cohorts. (D) Kaplan–Meier survival curves showing the survival probability for each group. The asterisk indicates a significant difference between groups at *p* < 0.05.

GL261 GBM tumors were easily discernible, displaying a higher signal intensity than the surrounding brain tissue on T_2_‐weighted images (Figure 1A). Tumor volume increased consistently in all cohorts (Figure 1C). At baseline, tumor volumes for the different groups were: saline (2.9 *±* 3.5 mm^3^), MN58b (3.1 *±* 2.6 mm^3^), TMZ (2.4 *±* 2.3 mm^3^), and MN58b + TMZ (2.8 *±* 1.9 mm^3^) (Table S1). No significant differences were observed in the baseline tumor volumes between groups (*p* > 0.05). After 6 days of treatment, tumor volume kept increasing in all groups: saline (15.8 *±* 17.7 mm^3^), MN58b (15.2 *±* 13.2 mm^3^), TMZ (17.5 *±* 16.7 mm^3^), and MN58b + TMZ (7.6 *±* 5.5 mm^3^). While insignificant differences were observed between groups, the MN58b + TMZ treatment group exhibited the lowest increase in percentage change (with respect to baseline) in mean tumor volume compared with the saline control and monotherapy groups, suggesting a trend toward reduced tumor progression (Figure 1C).

### 7.2 Survival

Survival probability for each group was estimated using Kaplan–Meier curves. Prolonged survival, in comparison to the saline‐treated group, was observed in the TMZ and combination treatment groups (Figure 1D). Specifically, ~85% of mice treated with TMZ alone and 100% of mice treated with the combination of MN58b and TMZ survived until the study endpoint (Day 6), suggesting a combinatorial effect by TMZ and MN58b. Despite the higher survival in the TMZ group, the difference in survival compared with the saline group was not statistically significant (*p* = 0.1). In contrast, a log‐rank test showed a significant difference between the MN58b + TMZ group and the saline control group (100% vs. 58%, *p* = 0.02, Figure 1D).

### 7.3 Assessment of Treatment‐Induced Metabolic Changes

**FIGURE 2:**
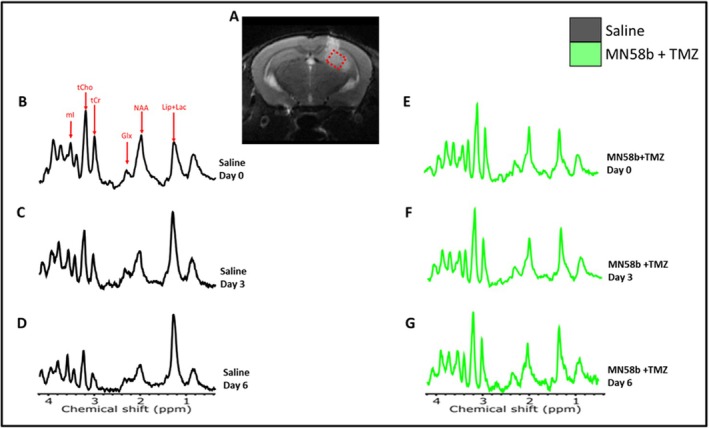
(A–G) T2‐weighted image (A) displaying the voxel position (highlighted region in dashed red) used for MRS acquisition within the tumor region. Representative MR spectra (B–G) from tumor‐bearing mice at baseline (Day 0), Day 3, and Day 6. Example spectra from a control mouse (B–D) and an MN58b + TMZ treated mouse (E–G) are shown. Resonances typically seen in the tumors have been labelled (Lip + Lac, NAA, Glx, tCr, tCho, and mI).

**FIGURE 3:**
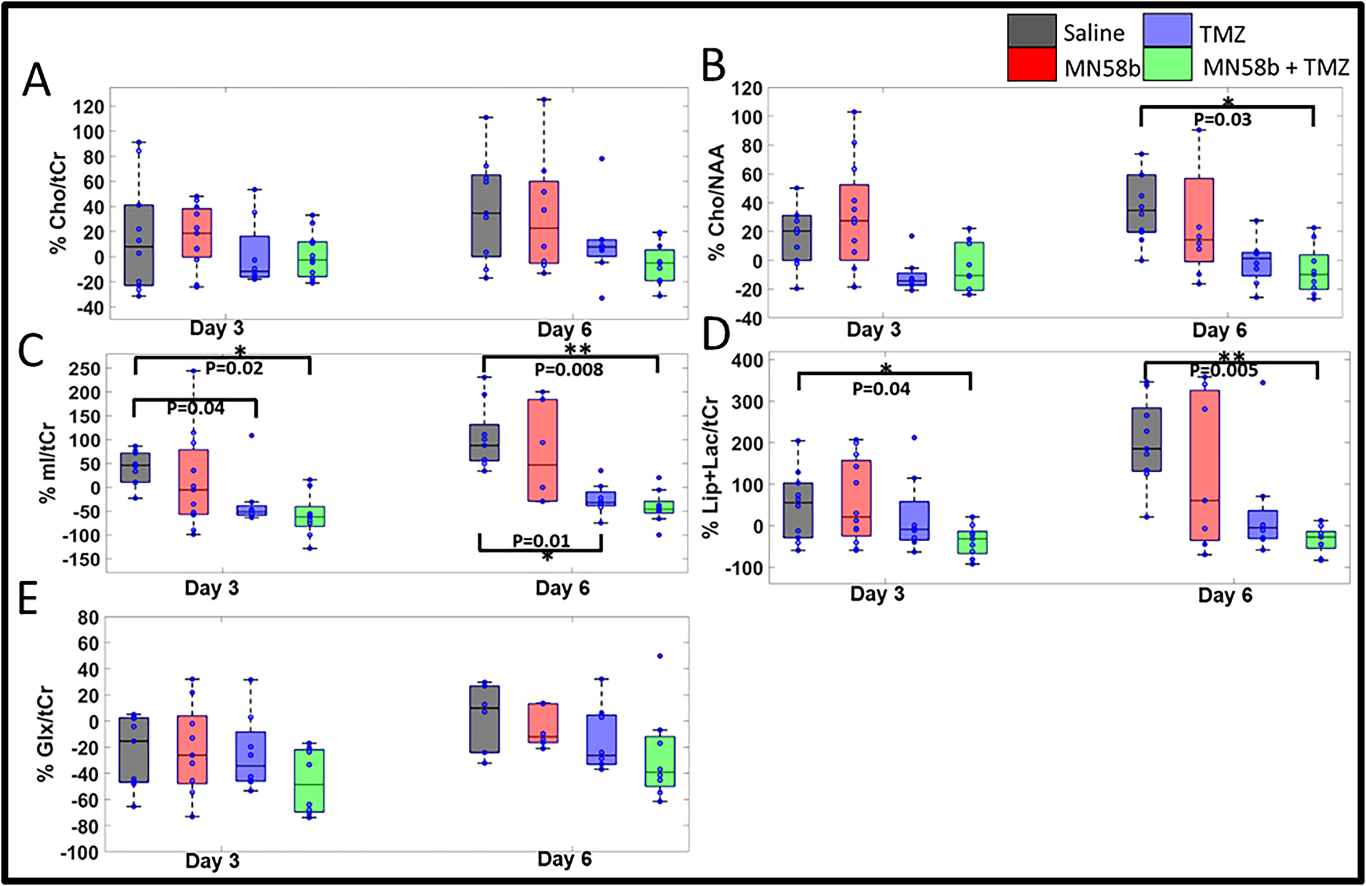
(A–E) Percentage change (relative to baseline) in metabolite ratios following drug treatment. (A) tCho/tCr, (B) tCho/NAA, (C) mI/tCr, (D) Lip + Lac/tCr, and (E) Glx/tCr. The asterisk indicates the difference between groups reached a significance level of *p* < 0.05.

Metabolic changes in response to different treatments were monitored using ^1^H MRS, with a PRESS voxel positioned on T_2_‐weighted images, as shown in Figure 2. By Day 3, no significant differences in tCho/tCr ratio across treatment groups were observed; however, by Day 6, the combination therapy group exhibited a 36% reduction in tCho/tCr ratio compared with the saline control group (1.59 *±* 0.26 vs. 2.28 *±* 0.69; *p* = 0.09, Table S1, Figure 3A). tCho/NAA ratios were also computed as they indicate neuronal integrity as an additional dimension, particularly relevant in GBM studies where NAA levels often decrease due to neuronal loss or damage. Similar to tCho/tCr, tCho/NAA was 28% and 26% lower in the TMZ‐alone and combination groups, respectively, compared with the control group (1.26 *±* 0.15 and 1.29 *±* 0.21 vs. 1.65 *±* 0.58) at Day 3. On Day 6, tCho/NAA was significantly lower in the combination group relative to the control group (48% lower; 1.24 *±* 0.21 vs. 1.92 *±* 0.50; *p* = 0.03, Table 1, Figure 3B). mI/tCr ratio was significantly lower in TMZ and TMZ + MN58b groups compared with the control group at Day 3 (0.97 *±* 0.39 and 0.82 *±* 0.27 vs. 1.50 *±* 0.64; *p* = 0.04 and 0.02, respectively), and Day 6 (1.08 *±* 0.22 and 0.90 *±* 0.20 vs. 2.37 *±* 1.20; *p* = 0.01 and 0.008, respectively, Table 1, Figure 3C). Lip + Lac/tCr ratio was also significantly lower in the TMZ + MN58b group compared with the control group on both Day 3 (1.80 *±* 0.60 vs. 3.71 *±* 1.81; *p* = 0.04) and Day 6 (1.82 *±* 0.52 vs. 7.13 *±* 2.3; *p* = 0.005, Table 1, Figure 3D), with a less pronounced decline in the MN58b‐alone group and a notable but non‐significant trend observed in the TMZ group. No significant changes in the Glx/tCr ratio were detected across all treatment cohorts relative to the control group at any time point (*p* > 0.05, Table S1, Figure 3E).

**TABLE 1:** Summary of key IVIM‐DWI and MRS parameters at baseline, Day 3, and Day 6. Data are presented as the mean ± standard deviation. The *p*‐value for the comparison with the control group is presented in brackets. The asterisk indicates the difference between groups where *p* < 0.05.

	Control	MN58b	TMZ	TMZ + MN58b
D (10^−3^ mm^2^/s)				
Day 0	1.35 ± 0.44	1.36 ± 0.41 (0.99)	1.52 ± 0.32 (0.52)	1.41 ± 0.46 (0.71)
Day 3	1.26 ± 0.21	1.35 ± 0.24 (0.79)	1.40 ± 0.27 (0.28)	1.46 ± 0.28 (0.26)
Day 6	1.25 ± 0.27	1.33 ± 0.23 (0.95)	1.51 ± 0.30 (0.24)	1.68 ± 0.32 (*0.03)
tCho/NAA				
Day 0	1.35 ± 0.29	1.18 ± 0.20 (0.26)	1.24 ± 0.19 (0.65)	1.31 ± 0.49 (0.95)
Day 3	1.65 ± 0.58	1.66 ± 0.41 (> 0.99)	1.26 ± 0.15 (0.76)	1.29 ± 0.21 (0.82)
Day 6	1.92 ± 0.50	1.72 ± 0.64 (0.92)	1.34 ± 0.19 (0.26)	1.24 ± 0.21 (*0.03)
mI/tCr				
Day 0	1.19 ± 0.54	0.92 ± 0.54 (0.78)	0.73 ± 0.24 (0.09)	0.60 ± 0.16 (0.08)
Day 3	1.50 ± 0.64	1.45 ± 0.85 (0.99)	0.97 ± 0.39 (*0.04)	0.82 ± 0.27 (*0.02)
Day 6	2.37 ± 1.20	1.85 ± 0.83 (0.73)	1.08 ± 0.22 (*0.01)	0.90 ± 0.20 (*0.008)
Lip + Lac/tCr				
Day 0	2.51 ± 1.16	2.07 ± 0.82 (0.72)	1.94 ± 1.27 (0.70)	1.72 ± 1.12 (0.78)
Day 3	3.71 ± 1.91	3.75 ± 2.01 (> 0.99)	2.90 ± 1.69 (0.81)	1.80 ± 0.60 (*0.04)
Day 6	7.13 ± 2.49	5.51 ± 3.62 (0.72)	3.58 ± 3.33 (0.09)	1.82 ± 0.52 (*0.005)
Mitotic index (Day 6)	7.40 ± 0.80	3.90 ± 0.65 (0.64)	4.10 ± 0.70 (0.75)	1.26 ± 1.50 (*0.01)
% apoptosis (Day 6)	0.63 ± 0.45	0.30 ± 0.14 (0.45)	0.24 ± 0.11 (0.34)	0.21 ± 0.13 (0.30)

Overall, these metabolic changes, particularly the reductions in the tCho/NAA, mI/tCr, and Lip + Lac/tCr, highlight the ability of MRS to capture early treatment‐induced metabolic alterations.

### 7.4 Changes in IVIM‐DWI Parameters Following Drug Treatment

**FIGURE 4:**
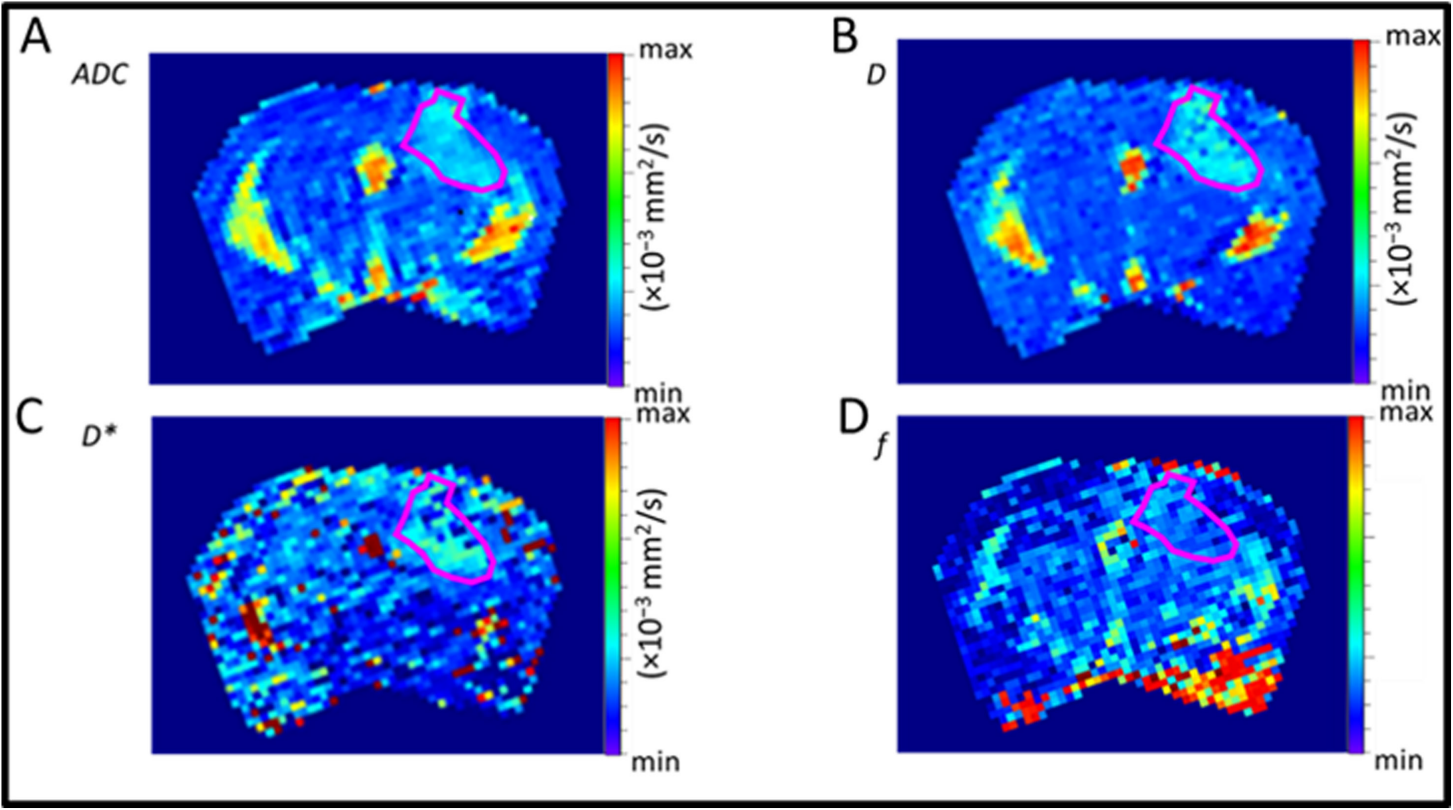
(A–D) Representative intravoxel incoherent motion (IVIM) parameter maps illustrating the apparent diffusion coefficient (ADC), pure diffusion coefficient (*D*), pseudo‐diffusion coefficient (D*), and perfusion fraction (*f*). The tumor region of interest (ROI) is delineated in magenta.

**FIGURE 5:**
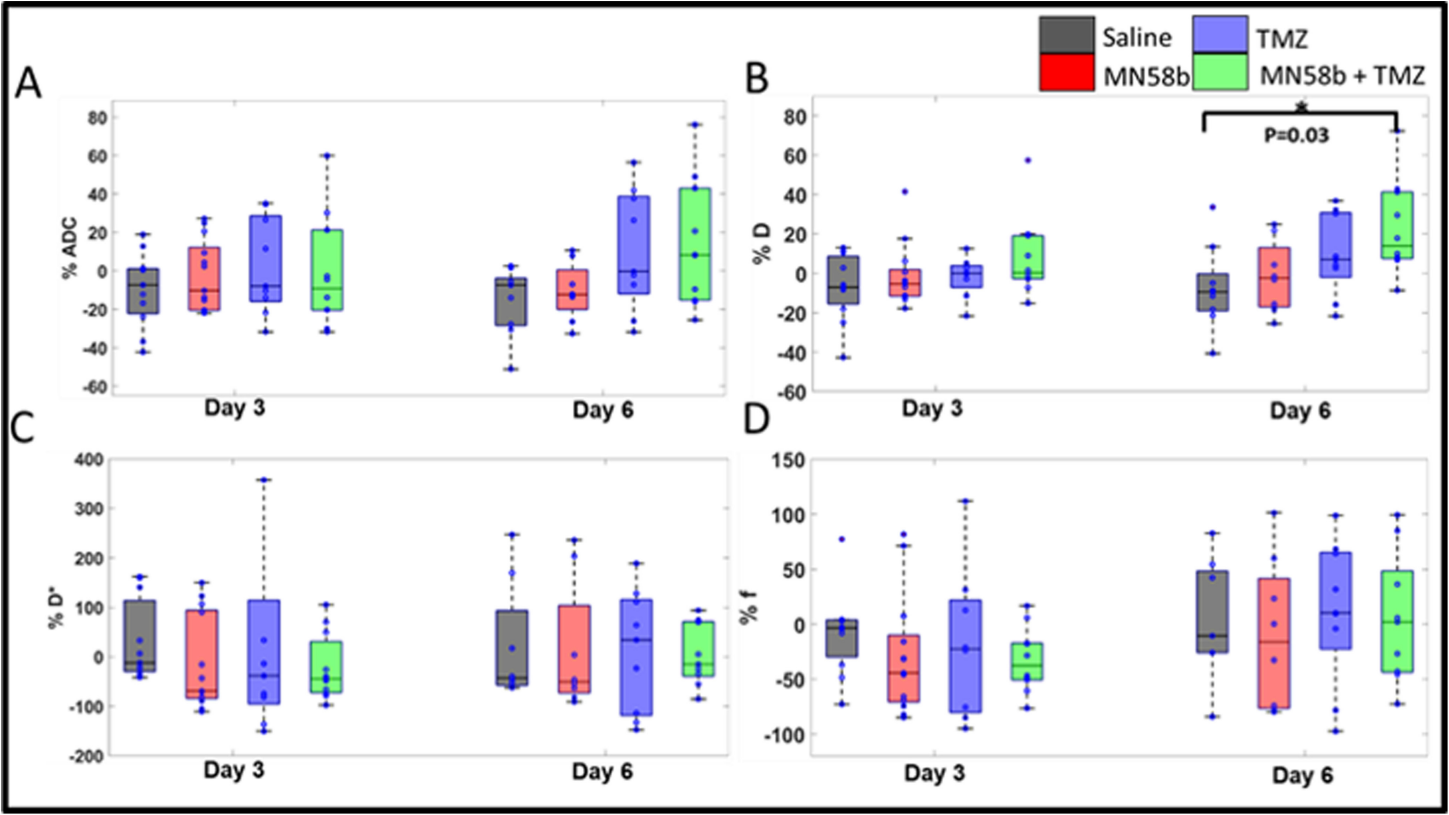
(A–D) Box plots illustrating the percent change in the IVIM‐DWI parameters, ADC, *D*, D*, and *f*, with time with respect to Day 0 for all groups. The asterisk indicates the difference between groups where *p* < 0.05.

Figure 4 shows representative parametric maps from the DWI data from a tumor bearing mouse including ADC (Figure 4A), *D* (Figure 4B), D* (Figure 4C) and *f* (Figure 4D). The red outline shows the ROI, which was used to calculate the individual parametric values. No significant differences in ADC values were observed between treatment groups and control group at any time point (*p* > 0.05); however, the MN58b + TMZ group exhibited an upward trend in ADC values by Day 6 compared with the other cohorts (Table S1, Figure 5A). Similarly, animals in treated groups demonstrated a trend of higher pure diffusion coefficient (*D*) values compared with the control group by Day 6, with MN58b + TMZ therapy group demonstrating significantly higher *D* values than the control group (1.68 *±* 0.32 *×* 10^*−*3^ mm^2^/s vs. 1.25 *±* 0.27 *×* 10^*−*3^ mm^2^/s; *p* = 0.03, Table 1, Figure 5B). No significant differences were detected in the pseudo‐diffusion coefficient (D*) or perfusion fraction (*f*) values across any of treatment groups compared with the saline control group at any time point (*p* > 0.05, Table S1, Figure 5C,D).

### 7.5 Histopathological Analysis

**FIGURE 6:**
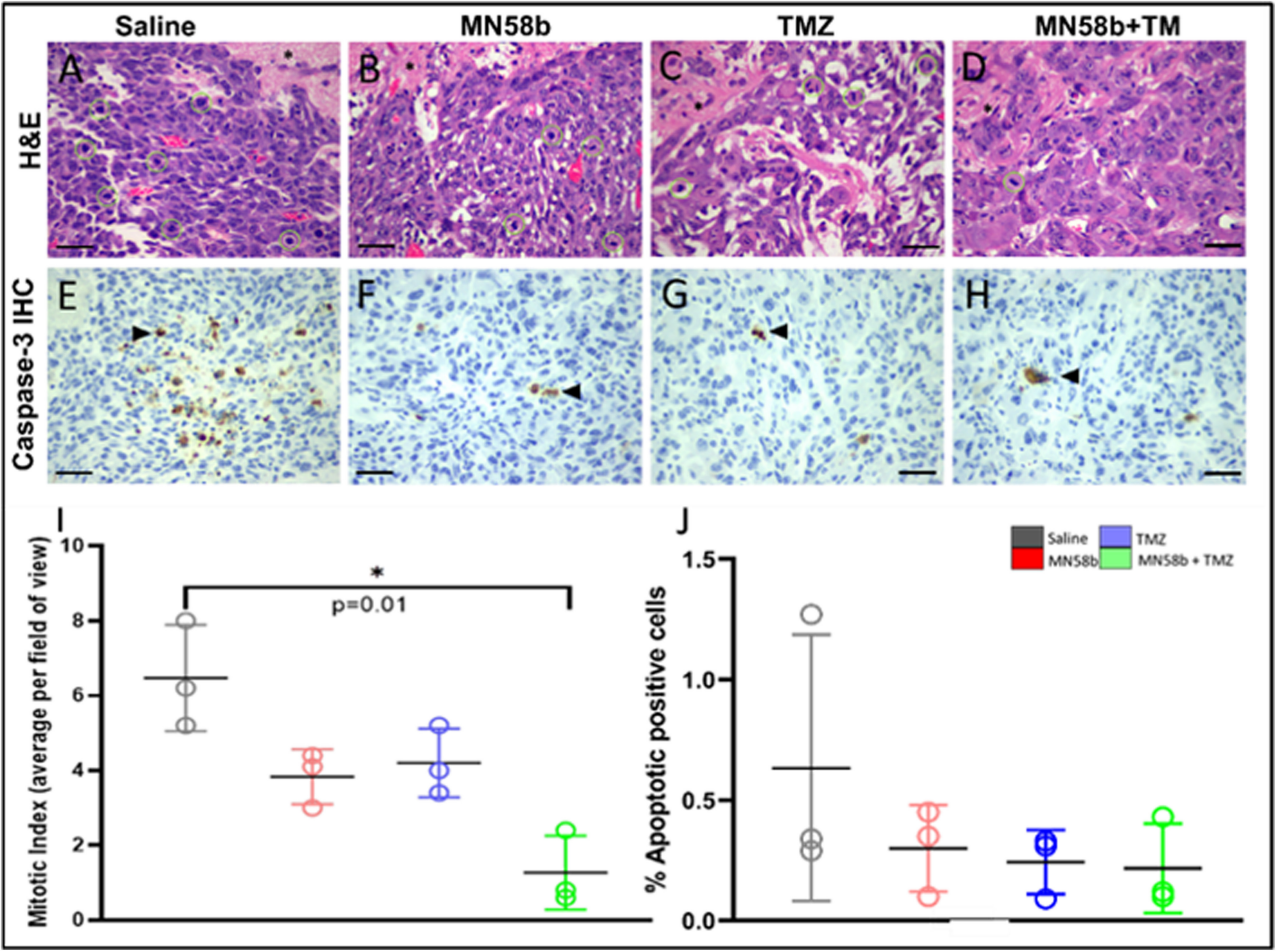
(A–J) Representative H&E images from the four experimental groups. (A–D) Representative high‐power fields close to the normal brain parenchyma (*) with highlighted mitotic figures (green circles) for saline (A), MN58b (B), TMZ (C), and MN58b + TMZ (D), hematoxylin–eosin, 400×. (E–H) Representative examples of positive cells for caspase‐3 expression (arrowhead) for saline (E), MN58b (F), TMZ (G), and MN58b + TMZ (H). Scalebars: 50 μm. Mitotic index (I) and percentage positive apoptotic cells (J) measurements were assessed from the tumor margins of three animals from each treatment group.

Histological analysis for proliferation (mitotic index, measured through H&E staining) and apoptosis (caspase‐3 expression) was performed after the final imaging time point (Day 6) on three animals from each group. Representative stains from each group of animals are shown in Figure 6A–H. While the mitotic index was significantly lower in the MN58b + TMZ therapy group compared with the saline group (1.26 *±* 1.50 vs. 7.40 *±* 0.80; *p* = 0.01, Table 1, Figure 6I), no significant differences were observed in the % caspase‐3 positive cells among any of the treated groups compared with the saline control group (*p* > 0.05, Table 1, Figure 6J).

## 8 Discussion

In this study, we observed combinatorial effects due to MN58b, a ChoK*α* inhibitor, and TMZ in an orthotopic GBM mouse model. The combination therapy resulted in significant metabolic and microstructural changes within the tumor, which were detectable using MRS and IVIM‐DWI prior to any observable changes in tumor volume. Specifically, the combination therapy demonstrated marked reductions in the tCho/NAA, mI/tCr, and Lip + Lac/tCr ratios relative to the control group, alongside a significant increase in D values, indicative of reduced tumor proliferation and lower cellularity. The combination therapy led to significantly increased survival compared with the control group.

Accurate and prompt diagnosis of tumor progression versus treatment response in patients with GBM is crucial for proper salvage therapy. MR techniques such as MRS and IVIM‐DWI effectively detect early treatment responses, which may be critical for effective treatment decisions. Importantly, tumor volume measurements, typically used in radiological assessments such as the response assessment in neuro‐oncology (RANO) criteria, lag behind treatment‐induced metabolic and microstructural changes. While RANO criteria provide a framework for assessing disease progression in brain tumors, they rely heavily on radiological measurements like tumor volume and contrast enhancement [26]. RANO criteria often fail to differentiate pseudo‐response (quick reduction in contrast enhancement without true tumoricidal effect) as well as pseudo‐progression (transient treatment‐related increase in contrast enhancement suggestive of treatment response) [5]. Pseudo‐progression is often associated with TMZ therapy. For example, metabolic changes detected by MR spectroscopy, such as alterations in choline‐containing compounds, have been used to differentiate true tumor progression from pseudoprogression in GBM patients, thereby guiding clinical management. These instances underscore the clinical relevance of metabolic imaging in informing treatment strategies [44]. Conversely, pseudo‐response is typically linked to anti‐angiogenic treatments, such as bevacizumab [6]. Although PET can aid in brain lesion assessment, its limited availability restricts clinical use [3, 4]. For example, the PET tracer L‐[methyl‐^11^C] methionine is effective in diagnosing recurrence but requires a cyclotron to synthesize the radio‐isotope [25]. A recent meta‐analysis reported that MR spectroscopy has the highest diagnostic accuracy, followed by MRI methods like DSC and DWI, outperforming traditional MR imaging in detecting treatment‐induced changes [25].

TMZ works by causing DNA methylation, which damages DNA, alters the cell cycle, and induces apoptosis, autophagy, and senescence [8]. Tumors expressing O6‐methylguanine‐DNA‐methyltransferase (MGMT) may resist TMZ‐induced cell death as MGMT repairs TMZ‐induced DNA methylation [7]. Even responsive tumors can develop resistance over time [7]. Research shows hypoxia supports TMZ resistance in GBM, while hyperoxia can sensitize tumors [11]. Hypoxia also contributes to resistance to anti‐angiogenic treatments, including bevacizumab, a vascular endothelial growth factor (VEGF) inhibitor [9]. Immunotherapies such as nivolumab (NIVO) have shown effectiveness against brain metastases from melanoma and increased survival in cancers like lung and kidney. However, NIVO combined with radiotherapy offers a lower survival benefit compared with TMZ combined with radiotherapy, making NIVO an inadequate substitute for TMZ [14]. Thus, there is an urgent need for new therapies to overcome TMZ‐related treatment resistance.

Choline is essential for cell membrane biosynthesis via the Kennedy pathway. Phosphocholine (PC) is a promising oncometabolite, which is dysregulated in many cancers, including endometrial, lung, breast, and prostate [17]. Abnormal choline metabolism, driven by ChoK*α* upregulation, is linked to tumor growth and drug resistance [19, 21]. ChoK*α*, the first enzyme in the Kennedy pathway, phosphorylates choline into PC, with elevated PC indicating tumor progression [17]. In brain tumors, higher ChoK*α* expression correlates with grade and malignancy [17, 20]. Targeting ChoK*α* shows promise in cancer therapies, with MRS‐based assessment and ChoK*α* inhibition yielding positive outcomes in breast cancer and glioblastoma models [17, 23, 24]. Our group has recently demonstrated reductions in perfusion, permeability, and increased necrosis in F98 tumors after treatment with JAS239, a 2nd generation ChoK*α* inhibitor [44]. A previous study from our group also reported that JAS239 reduced tCho concentrations and tCho/tCr and tCho/NAA ratios in rodent GBM models, indicating metabolic changes in response to ChoK*α* inhibition [24]. ChoKα inhibition by MN58b disrupts phosphatidylcholine synthesis, which alters membrane lipid composition and cellular stress. This metabolic stress may impair DNA repair mechanisms, potentially sensitizing tumor cells to the DNA‐damaging effects of TMZ. Thus, combining MN58b and TMZ could enhance therapeutic efficacy by simultaneously targeting membrane metabolism and DNA integrity [22, 23, 24]. The tCho/NAA ratio is a recognized biomarker for tumor proliferation and malignancy in glioblastoma, with elevated tCho levels typically indicating increased membrane turnover in rapidly growing tumors [17, 20]. In this study, by Day 6, the TMZ + MN58b group demonstrated significantly lower tCho/NAA as well as increased survival compared with the control group, suggesting an effective inhibition of tumor proliferation via cell cycle arrest, potentially due to the combined therapeutic effects of MN58b + TMZ on cell cycle progression. MN58b has been shown to inhibit ChoKα's catalytic activity but does not significantly interfere with its protein stability in all models [45]. While MN58b is a known ChoKα inhibitor, its effect on tCho levels can vary depending on the tumor model, drug dosage, and degree of ChoKα inhibition achieved. In our study, the absence of a significant reduction in tCho/tCr or tCho/NAA in the MN58b monotherapy group may be due to compensatory metabolic adaptations, subthreshold pharmacodynamic effects at the examined time point, or tumor heterogeneity. Our previous study reported such an observation where the GL261 mouse model exhibited a modest effect in response to JAS239 given at the same treatment regimen of MN58b, indicating the aggressive nature of the GL261 mouse model [24]. In our study, the tCho/NAA reduction observed in the combination group may reflect partial recovery of surrounding neuronal integrity or differential effects on NAA relative to tCho. The lack of change in tCho/tCr may imply that Cr levels remained stable or were slightly elevated, offsetting any subtle tCho reduction. A lower mI/tCr ratio in the combination therapy group on Days 3 and 6 suggests decreased astrocytic activity and increased cellular stress within the TME. This is consistent with prior research, which indicated treatment‐induced disruption of metabolic pathways linked to glial proliferation and stress responses in GBMs [24, 46, 47]. The resonance at ~3.56 ppm includes contributions from both myo‐inositol and glycine. While myo‐inositol is associated with glial activity and osmoregulation, glycine levels are elevated in tumors and correlate with proliferation. The observed decrease of the peak at 3.56 ppm may thus also reflect a reduction in glycine due to the anti‐proliferative effects of the drugs. Future studies using both short and long echo time MRS could help differentiate between these metabolites and better describe their contribution to treatment response assessment. These would also aid in better differentiation of lactate from mobile lipids and macromolecules. The significant reduction in Lip + Lac/tCr ratio in the combination therapy group was observed as early as Day 3 and persisted through Day 6, suggesting reduced glycolytic activity and lipid turnover and storage due to effective therapeutic action, as reported earlier [48]. The observed decrease may be due to a reduction in lipid and glycolytic metabolism because the PRESS spectroscopy sequence used in this study cannot differentiate between the overlapping resonances of lipids from lactate. For future studies, utilizing a longer echo time in MRS or lactate‐edited pulse sequences may aid in differentiating lactate and lipid signals, potentially allowing for a clearer assessment of treatment response and metabolic shifts within the tumor [49]. Subramani et al. observed that TMZ treatment reduced the ^13^C MRS‐detectable pyruvate‐to‐lactate conversion in wild‐type IDH glioma but did not affect ^1^H MRS‐detectable Glx (glutamine and glutamate). In contrast, mutant IDH1 tumors demonstrated increased Glx in response to TMZ [50]. Similarly, Radoul et al. reported elevated Glx following the administration of IDH1 inhibitors in IDH1‐mutant glioma models [51]. However, Bhaduri et al. found no significant change in Glx in GBM models treated with a CKI [24]. In our study, the Glx/tCr ratio remained unchanged across treatment groups, suggesting that changes in Glx levels may be treatment or cell‐line‐specific.

In our study, *D* values were significantly elevated in the TMZ + MN58b group, indicating lower cellularity and increased motion of water molecules in the treatment group, suggesting a successful therapeutic response. This observation aligns with findings from prior studies in various cancers, where increased *D* values have been linked to treatment response, including head‐and‐neck cancer, brain metastases, and hepatocellular carcinoma [52, 53, 54]. While ADC values also demonstrated an upward trend in the combination therapy group in our study, this change was not as significant as the increase observed in *D*. These results highlight the sensitivity of IVIM‐DWI derived *D* as a marker for cellularity changes in response to treatment, offering a more accurate assessment of therapeutic impact than ADC. No significant changes in perfusion‐related IVIM‐DWI parameters D* and *f* were observed across all treated groups, consistent with the literature [52, 53, 54]. This suggests that MN58b and TMZ, either alone or in combination, did not significantly impact tumor vasculature. In contrast, antiangiogenic treatments such as bevacizumab significantly reduce D* and *f* values in glioma models, highlighting the potential role of combining vascular‐targeted therapies with metabolic inhibitors like MN58b for better therapeutic outcomes [55]. In this study, IVIM‐DWI was chosen for its ability to capture both diffusivity and perfusion fraction, providing combined microstructural and vascular characterization within a single acquisition protocol. However, because only microstructural changes were detected, future studies could benefit from exploring complementary diffusion techniques such as DTI and DKI, which offer more detailed microstructural metrics, including anisotropy and kurtosis, particularly at higher b‐values, to further characterize tissue heterogeneity. Additionally, the potential value of incorporating other endogenous contrast MRI methods, such as ASL, could be investigated alongside IVIM‐DWI to provide a non‐invasive complement or alternative for a more comprehensive assessment of tumor vasculature.

Our imaging‐based findings were further supported by the reduced mitotic index in the combination therapy group, which agrees with prior studies [24, 49]. However, we did not observe any significant changes in the degree of apoptosis (caspase‐3) in the combination treatment group, indicating an absence of marked apoptosis due to the treatment. This probably indicates an induced cytostatic effect (cell cycle arrest) rather than direct cytotoxicity (apoptosis), which was also reported in tumors treated with TMZ [56] and a CKI [57]. We do acknowledge that our observations are based on only three samples per group, as we were unable to process additional samples due to increased cost as well as using the tumor tissue for another study.

Although promising, this study has certain limitations. Firstly, the dose regimen used for MN58b may not have been optimal for the GL261 model, as it was chosen based on previous studies on breast cancer [54] and a rat model [23] of GBM. Second, the aggressive nature of the GL261 tumor model necessitated a short imaging window, limiting the ability to assess the longer‐term effects of the combination therapy.

Future studies should focus on optimizing the dosing regimen of MN58b and exploring its efficacy as a monotherapy or in combination with other treatments like radiotherapy or additional targeted therapies like anti‐angiogenic agents and glycolytic inhibitors like lonidamine [58]. Expanding the imaging window could provide more robust insights into the therapeutic effects of the treatments. While incorporating complementary imaging techniques such as DCE‐MRI would provide a more comprehensive understanding of changing tumor perfusion dynamics during therapy, ASL may serve as a useful complement or alternative to IVIM for assessing vascular characteristics, particularly when avoiding contrast agents is desirable. These data were obtained from chemically induced rodent GBM cell lines, which rapidly form large tumors, and thus, extrapolation to the human context should be approached with caution. In future studies, the use of human‐derived xenograft models (e.g., U251) could be considered, as they may better reflect the time course of tumor formation and the therapy resistance observed in human GBM. However, PDX models have their own limitations as they are grown in immune‐deficient rodents, and thus the tumor‐immune cell interaction or the TME observed in human tumors cannot be truly replicated in these models either.

## 9 Conclusion

^1^H MRS and IVIM‐DWI may be valuable non‐invasive tools for monitoring treatment‐induced changes in tumors, providing insights into metabolic, diffusion, and perfusion characteristics that report on the effectiveness of cancer treatments. Targeting ChoK*α* and inducing DNA damage in tumor cells with TMZ offers a promising dual therapeutic strategy for glioblastoma treatment. As both drugs are sufficiently bioavailable within the GBM to have a measurable impact and target disparate, unrelated cellular targets, this approach can potentially enhance treatment and reduce drug resistance of the notoriously TMZ‐resistant GBM.

## Conflicts of Interest

The authors declare no conflicts of interest.

## Supporting information


**Data S1:** Supporting information.

**Figure S1:** A representative ^1^H MRS spectrum illustrating the fitting performance from a 9L tumor‐bearing rat (original spectra in blue, fitted spectra in red, and residual in black). This figure is from on our previously published work by Bhaduri et al. [25], which used the same acquisition and quantification pipeline for GL261 glioma models reported in this paper.

**Figure S2:** Examples of the digital analysis workflow for caspase‐3: (A) Low power magnification of tumor (*) surrounded by normal parenchyma (**) identified as region of interest (ROI—yellow line). Scalebar: 800 μm. (B) High power magnification of the segmentation mask for caspase‐3 negative (blue) and positive (red) cells. Scalebar: 50 μm.

**Table S1:** Summary of non‐significant parameters at baseline, Day 3, and Day 6. Data are presented as the mean ± standard deviation. The *p*‐value for the comparison with the control group is presented in brackets.

## Data Availability

The data that support the findings of this study are available from the corresponding author upon reasonable request.
