# Supplementary material for: Evaluating the Combined Effect of a Choline Kinase Inhibitor and Temozolomide Therapy in a Mouse Model of Glioblastoma Using 1H MR Spectroscopy and IVIM‐DWI
Source: NMR Biomed. 2025 Aug 4;38(9):e70113. doi: 10.1002/nbm.70113 (PMC12319478; doi:10.1002/nbm.70113)
Supplement: Supplementary file 1 — Data S1: Supporting information. [file NBM-38-e70113-s001.docx]

**Evaluating the combined effect of a choline kinase inhibitor and temozolomide therapy in a mouse model of glioblastoma using** 1 **H MR spectroscopy and IVIM-DWI**

**Tareq Alrashidi1, Sourav Bhaduri2,3, Elisabeth Non Gash1, Mohesh Moothanchery1, Christopher Ball1, Mahon L Maguire1, Lorenzo Ressel4 and Harish Poptani1***†

*1Centre for PreClinical Imaging, University of Liverpool, Liverpool, United Kingdom*

*2Institute for Advancing Intelligence (IAI), TCG CREST, Kolkata, India*

*3Academy of Scientific and Innovative Research (AcSIR), Ghaziabad 201002, India*

*4Department of Veterinary Anatomy Physiology and Pathology, University of Liverpool, Chester, United Kingdom*

*Corresponding Author Email: [harish.poptani@liverpool.ac.uk](mailto:harish.poptani@liverpool.ac.uk)

Supplementary TABLE 1

|  | **Control** | **MN58b** | **TMZ** | **TMZ+MN58b** |
| --- | --- | --- | --- | --- |
| **Tumour volume (mm^3^)** | | | | |
| **Day 0** | 2.87±3.67 | 2.97±2.67 (0.99) | 2.35±2.42 (0.92) | 2.85±1.98 (0.99) |
| **Day 3** | 8.33±8.26 | 6.71±6.58 (0.94) | 7.82±7.13 (0.97) | 3.96±1.91 (0.40) |
| **Day 6** | 15.8±16.2 | 16.1±14.1 (0.98) | 17.5±17.4 (0.99) | 7.63±5.58 (0.48) |
| **tCho/tCr** | | | | |
| **Day 0** | 1.60±0.66 | 1.62±0.29 (0.99) | 1.54±0.36 (0.93) | 1.52±0.27 (0.91) |
| **Day 3** | 1.84±0.69 | 1.49±0.40 (0.97) | 1.67±0.42 (0.91) | 1.63±0.23 (0.80) |
| **Day 6** | 2.28±0.65 | 2.18±0.72 (0.96) | 1.92±0.57 (0.75) | 1.59±0.24 (0.09) |
| **Glx/tCr** | | | | |
| **Day 0** | 0.65±0.15 | 0.57±0.23 (0.26) | 0.41±0.10 (0.65) | 0.42±0.10 (0.95) |
| **Day 3** | 0.51±0.16 | 0.52±0.19 (0.99) | 0.55±0.13 (0.76) | 0.49±0.12 (0.82) |
| **Day 6** | 0.68±0.17 | 0.62±0.12 (0.92) | 0.59±0.19 (0.26) | 0.56±0.11 (0.14) |
| **ADC (10^−3^ mm^2^/s)** | | | | |
| **Day 0** | 1.52±0.50 | 1.59±0.46 (0.98) | 1.48±0.36 (0.96) | 1.54±0.37 (0.98) |
| **Day 3** | 1.36±0.29 | 1.48±0.27 (>0.99) | 1.39±0.37 (0.92) | 1.48±0.60 (0.95) |
| **Day 6** | 1.28±0.26 | 1.39±0.20 (0.94) | 1.63±0.49 (0.19) | 1.79±0.52 (0.10) |
| **D* (10^−3^ mm^2^/s)** | | | | |
| **Day 0** 36.2±26.7 26.1±22.4 (0.91) 15.3±9.88 (0.52) 29.5±18.5 (0.97)  **Day 3** 47.3±28.4 29.2±24.1 (0.65) 47.7±34.1 (0.97) 26.8±17.2 (0.48)  **Day 6** 44.1±36.2 39.7±30.5 (0.93) 38.1±31.8 (0.95) 37.3±17.8 (0.92) | | | | |
| **f** | | | | |
| **Day 0**  **Day 3**  **Day 6** | 0.13±0.09  0.14±0.08  0.14±0.07 | 0.10±0.05 (0.58) 0.10±0.03 (0.60) 0.12±0.06 (0.94) | 0.11±0.04 (0.67) 0.12±0.06 (0.90) 0.14±0.07 (0.96) | 0.14±0.05 (0.96) 0.10±0.03 (0.38) 0.13±0.07 (0.92) |

Summary of non-significant parameters at baseline, day 3, and day 6. Data are presented as mean and ± standard deviation. The p-value for the comparison with the control group is presented in brackets.


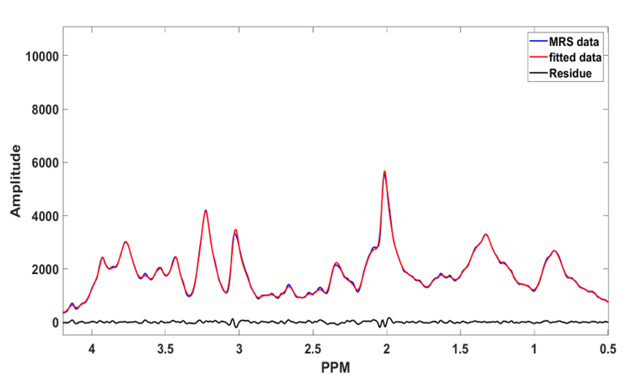


**Supplementary Figure 1:** A representative ^1^H MRS spectrum illustrating the fitting performance from a 9L tumour-bearing rat (original spectra in blue, fitted spectra in red, and residual in black). This figure is from on our previously published work by Bhaduri et al. [25], which used the same acquisition and quantification pipeline for GL261 glioma models reported in this paper.

**
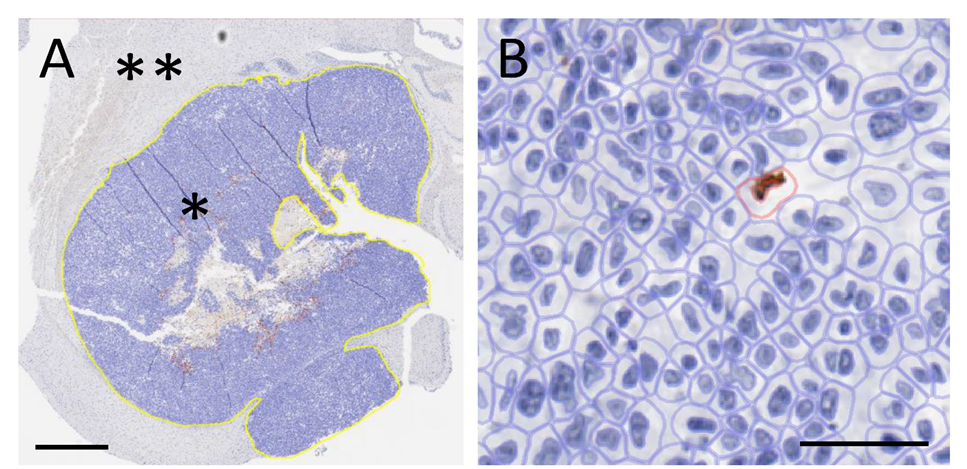
****Supplementary Figure 2:** Examples of the Digital analysis workflow for Caspase-3: **A**: Low power magnification of tumour (*) surrounded by normal parenchyma (**) identified as region of interest (ROI -Yellow line). Scalebar: 800 microns. **B**: High power magnification of the segmentation mask for Caspase-3 negative (blue) and positive (red) cells. Scalebar: 50 microns.
