# Supplementary figures and images for: Evaluating the Combined Effect of a Choline Kinase Inhibitor and Temozolomide Therapy in a Mouse Model of Glioblastoma Using 1H MR Spectroscopy and IVIM‐DWI
Source: NMR Biomed. 2025 Aug 4;38(9):e70113. doi: 10.1002/nbm.70113 (PMC12319478; doi:10.1002/nbm.70113)

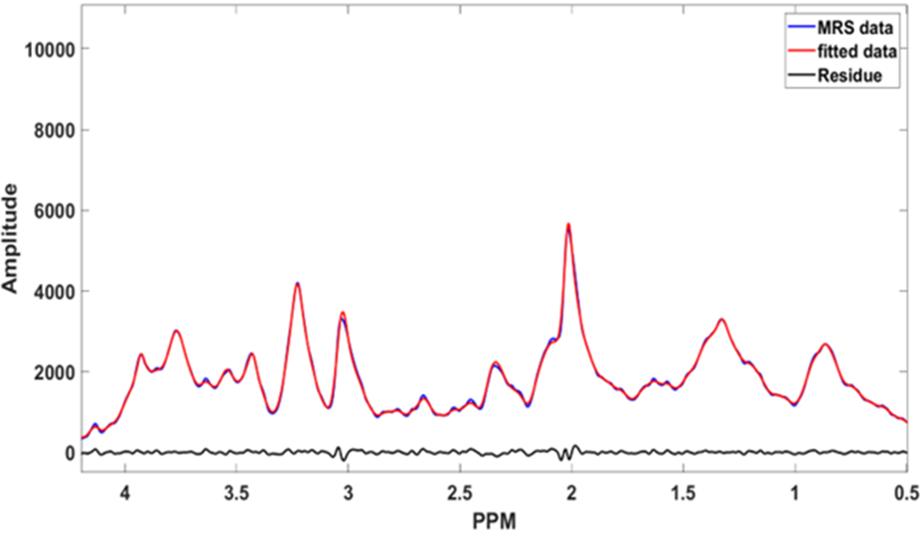

Supplement: Supplementary file 2 — Figure S1: A representative 1H MRS spectrum illustrating the fitting performance from a 9L tumor‐bearing rat (original spectra in blue, fitted spectra in red, and residual in black). This figure is from on our previously published work by Bhaduri et al. [25], which used the same acquisition and quantification pipeline for GL261 glioma models reported in this paper. [file NBM-38-e70113-s004.tiff]

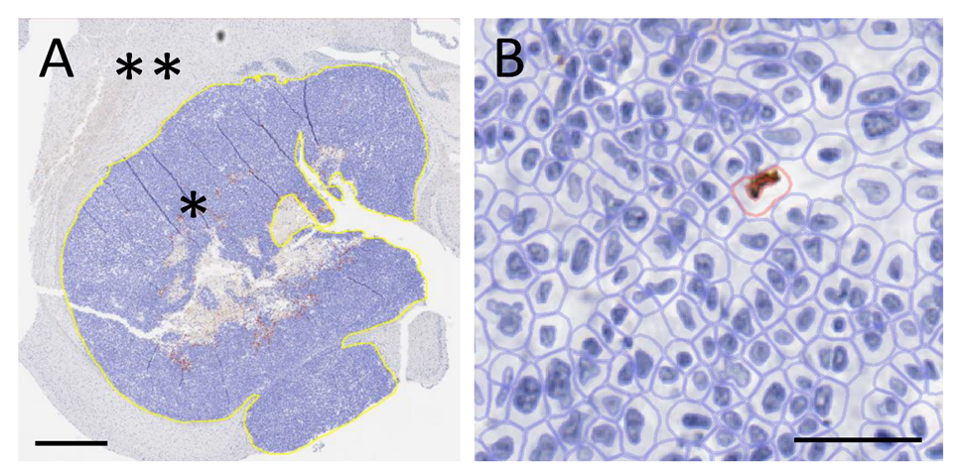

Supplement: Supplementary file 3 — Figure S2: Examples of the digital analysis workflow for caspase‐3: (A) Low power magnification of tumor (*) surrounded by normal parenchyma (**) identified as region of interest (ROI—yellow line). Scalebar: 800 μm. (B) High power magnification of the segmentation mask for caspase‐3 negative (blue) and positive (red) cells. Scalebar: 50 μm. [file NBM-38-e70113-s003.tiff]
